# Synthesis and Characterization of Multifunctional Chitosan-MnFe_2_O_4_ Nanoparticles for Magnetic Hyperthermia and Drug Delivery

**DOI:** 10.3390/ma3074051

**Published:** 2010-07-13

**Authors:** Dong-Hyun Kim, David E. Nikles, Christopher S. Brazel

**Affiliations:** 1 Department of Chemical and Biological Engineering, Box 870203, The University of Alabama, Tuscaloosa, AL 35487-0203 USA; E-Mail: dhkim0405@gmail.com (D.-H.K.); 2 Department of Chemistry and Center for Materials for Information Technology, Box 870336, The University of Alabama, Tuscaloosa, AL 35487 USA; E-Mail: dnikles@mint.ua.edu (D.N.)

**Keywords:** multifunctional magnetic nanoparticles, chitosan, MnFe_2_O_4_ nanoparticles, magnetic hyperthermia, drug delivery

## Abstract

Multifunctional nanoparticles composed of MnFe_2_O_4_ were encapsulated in chitosan for investigation of system to combine magnetically-triggered drug delivery and localized hyperthermia for cancer treatment with the previously published capacity of MnFe_2_O_4_ to be used as an efficient MRI contrast agent for cancer diagnosis. This paper focuses on the synthesis and characterization of magnetic MnFe_2_O_4_ nanoparticles, their dispersion in water and their incorporation in chitosan, which serves as a drug carrier. The surface of the MnFe_2_O_4_ nanoparticles was modified with meso-2,3-di-mercaptosuccinic acid (DMSA) to develop stable aqueous dispersions. The nanoparticles were coated with chitosan, and the magnetic properties, heat generation and hydrodynamic size of chitosan-coated MnFe_2_O_4_ were evaluated for various linker concentrations and in a range of pH conditions.

## 1. Introduction

Recently, interest in the use of magnetic nanoparticles (MNPs) for biomedical applications has increased due to their unique multifunctional properties. When the size of the magnetic particles decreases to below a critical size, generally less than 15 nm [1], each nanoparticle becomes a single magnetic domain and exhibits remarkable superparamagnetic behavior similar to paramagnetism. Each individual nanoparticle has a large constant magnetic moment and behaves like a giant paramagnetic atom with a fast response to applied magnetic fields with negligible remanence and coercivity. These features make superparamagnetic nanoparticles attractive for a wide range of biomedical applications because the magnetization can be controlled by an external magnetic field without the risk of magnetic attraction at room temperature. By coating MNPs with a polymer, drug molecules can also be sequestered in the same device. Therefore, MNPs have been used for magnetic hyperthermia, triggered or localized drug delivery, magnetic resonance imaging and biosensors [2,3,4,5].

MNPs can be designed to heat when a high frequency magnetic field is applied. The heating, which has found uses in hyperthermia cancer treatment [6,7], is related to losses during the process of magnetization reversal within the particles. However, good control and optimization of the heating is needed to reach and maintain temperatures around 42 °C. By combining MNPs with targeting moieties, heating to thermal ablation temperatures may also be feasible. The design of MNPs with efficient heating profiles can minimize the quantity needed to reach these effective temperatures. Furthermore, including chemotherapy drugs with targeted MNPs may provide effective localized combination therapy, particularly for polymer membranes made of LCST polymers with imbedded magnetite which have been shown to deliver fluorescein in a pulsatile fashion [8] or block copolymer micelles coated around gold or magnetic nanoparticles which show a squeezing effect in their hydrodynamic size with an increase in temperature [9].

MnFe_2_O_4_ MNPs have a greater biocompatibility [10] and a strong T_2_ phase contrast for magnetic resonance imaging [11,12] compared with Fe_3_O_4_, γ-Fe_2_O_3_, CoFe_2_O_4_, and NiFe_2_O_4_ MNPs. Thus, MnFe_2_O_4_ MNPs can provide the multiple functionalities of imaging, hyperthermia and triggered drug release. The synthesis of monodisperse nanoparticles of Fe_3_O_4_, γ-Fe_2_O_3_ and MFe_2_O_4_ (where M=Co, Ni or Mn) developed by high temperature organic solution phase methods for have been produced and dispersed in various organic solvents [13]. The magnetic susceptibility of MnFe_2_O_4_ nanoparticles is higher than for other ferrite nanoparticles such as Fe_3_O_4_, CoFe_2_O_4_ and NiFe_2_O_4_ with magnetic spins of 5 μ_b_ [11]. However, a ligand exchange step is required to develop stable aqueous dispersions of MNPs for biomedical applications. These ligands may also facilitate conjugation with targeting biomolecules, drugs or functional polymers.

Coating MNPs with a biopolymer allows sequestration of drug molecules, improves biocompatibility and can offer chemistries to allow attachment of targeting moieties. Chitosan, a naturally-occurring polysaccharide, is used frequently in drug delivery and biomaterials applications. The primary amine groups in chitosan render special attributes for applications in biomedicine. Chitosan has a net positive charge and is mucoadhesive [14], and has been found to be biocompatible with living tissue since it does not cause allergic reactions and rejection [15], and has been proven safe in rats when given at up to 10% in the diet [16]. Chitosan can also be easily crosslinked and modified into gels which can carry biologically-active molecules. Thus, chitosan has been used in conjunction with magnetic particles for magnetic drug targeting [17,18,19,20,21]. This technology allows localized delivery as the coated MNPs concentrate drugs in the region where a constant, externally-applied magnetic field is applied. The synthesis of chitosan-coated MNPs has been shown using ionic gelation or using a crosslinking agent (glutaraldehyde) after making MNPs by coprecipitation [22,23]. However, these techniques usually yield micron-sized chitosan-coated MNPs.

Magnetic heating can be effectively achieved using an AC magnetic field. Heating is due to two different loss processes (Néel and Brownian relaxation). For superparamagnetic nanoparticles, the power loss is calculated from [24]:

(1) $$P={\left(mH\omega \tau_{eff}\right)}^{2}/[2\tau_{eff}kT\;V(1+\omega^{2}{\tau_{eff}}^{2})]$$

where m is the MNP magnetic moment, ω the field frequency, k is the Boltzmann constant, T is absolute temperature, H is the AC magnetic field amplitude, V is the nanoparticle volume and τ_eff_ is the effective relaxation time. When an AC magnetic field is applied to MNPs, their magnetic orientations attempt to align in the direction of the magnetic field. After the magnetic field is switched off, the total magnetization disappears due to the statistical random reorientation of the nanoparticles with an effective relaxation time (τ_eff_ ) that is a combination of Brownian relaxation (τ_B_) and Néel relaxation (τ_N_) [25]:

(2) $$\tau_{eff}=\frac{\tau_{B}\tau_{N}}{\tau_{B}+\tau_{N}}$$

The Brownian relaxation time constant can be found by:

(3) $$\tau_{B}=3\eta V_{H}/kT$$

where η is the carrier fluid viscosity, and V_H_ is the hydrodynamic volume of the MNP. Néel relaxation is given by:

(4) $$\tau_{N}=\tau_{0}\exp(KV/kT)$$

where τ_0_ is on the order of 10^-9^ s, K is the anisotropy constant of the MNP, and V is the its volume.

The power loss equation (1) shows that magnetic field heating is a function of the material, MNP size, and the field characteristics. From equation (3), the effect of chitosan coating in MnFe_2_O_4_ MNPs on the heat generation could be described from the Brownian relaxation which depends on viscosity and hydrodynamic size. If the size of polymer coated MNPs increased above a critical size, Brownian relaxation dominates the effective relaxation time. However, for cases such as this experimental investigation, where the nanoparticles are quite small (12 nm) and they are constrained by the presence of a polymeric surface coating, heat generation in chitosan-MnFe_2_O_4_ MNPs is expected to depend on the Néel relaxation time. A recent study by Fortin *et al.* [26] has investigated the contributions of Brownian and Néel relaxation to the heating of maghemite (primarily Néel) and cobalt ferrite (primarily Brownian) MNPs, so the exact contributions to heating can vary, depending on the viscosity of the NP microenvironment.

In this study, multifunctional chitosan-MnFe_2_O_4_ MNPs were synthesized and characterized for hyperthermia and drug release behavior.

## 2. Experimental Section

### 2.1. Materials

Absolute ethanol and hexane were purchased from Fisher Scientific (Fair Lawn, NJ) and benzyl ether, 1,2-hexadecanediol, oleic acid, oleylamine, manganese(II) acetylacetonate, iron(III) acetylacetonate and chitosan (91.8 % deacetylation, low molecular weight; Brookfield viscosity of 36 cp for a 1 wt % acetic acid solution) were purchased from Aldrich Chemical Co (Milwaukee, WI). Meso- 2,3-dimercaptosuccinic acid (DMSA) and dimethyl sulfoxide (DMSO) were obtained from Acros Organics (Pittsburgh, PA) and Fisher Scientific (Pittsburgh, PA), respectively. EDC (1-ethyl-3-(3-dimethylaminopropyl) carbodiimide hydrochloride) was purchased from Pierce Biotechnology (Rockford, IL). Unless otherwise noted, all chemicals were used as received without further purification.

### 2.2. Forming Water-Dispersible MnFe_2_O_4_ Nanoparticles

MnFe_2_O_4_ nanoparticles were synthesized using the seed mediated growth method [27]. Seeds of MnFe_2_O_4_ nanoparticles were prepared in a high-temperature organic solution in a three-neck 50 mL round bottom flask (Kontes) fitted with a 150 mm condenser, a thermocouple and a rubber septum. The top of the condenser was fitted with a system to provide nitrogen flow into and out of the condenser to maintain an inert atmosphere. 2 mmol of Fe(acac)_3_, 1 mmol of Mn(acac)_2_, 10 mmol of 1,2-hexadecanediol and benzyl ether (20 mL) were mixed and stirred under nitrogen. Oleic acid (2 mmol) and oleylamine (2 mmol) were then injected into the mixture. A 40 mg seed of MnFe_2_O_4_ MNPs dispersed in hexane (3 mL) was added. It was then heated to 100 °C for 30 min to remove the hexane, followed by heating to 200 °C for 1 h. The solution was then refluxed for 1 h at 298 °C. The black-brown mixture was cooled to room temperature, and ethanol was added to precipitate MNPs, followed by centrifugation to remove solvent, and redispersion in hexane.

A ligand exchange reaction was used to introduce the DMSA ligand that renders the MNPs water-soluble. Specifically, the oleic acid/oleylamine-MnFe_2_O_4_ MNPs were suspended in hexane at 10 (w/v) % and DMSA also was dissolved in DMSO at 10 (w/v) %. These two solutions were mixed at a 1:1 volume. After 6 hours of sonication, the hexane layer (containing any remaining organic precursors) was discarded yielding water-soluble DMSA-MnFe_2_O_4_ MNPs dissolved in DMSO. The MNPs were purified using at least five DI water washes with magnetic separation of particles from the supernatant.

### 2.3. Chitosan-coated MnFe_2_O_4_ Nanoparticles

**Figure 1:**
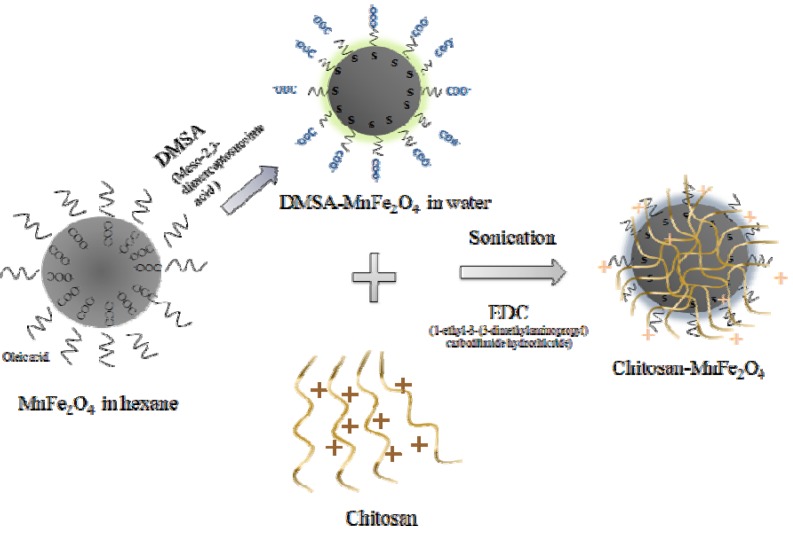
Schematic of the synthesis of chitosan-MnFe_2_O_4_ MNPs.

The synthetic process for coating MnFe_2_O_4_ nanoparticles with chitosan is illustrated in Figure 1. Here, a 1% (w/v) chitosan solution was prepared by dissolving chitosan (0.2 g) in a 2.0 % aqueous acetic acid solution for 4 h. 3.0 mL of aqueously-dispersed DMSA-MnFe_2_O_4_ MNPs (10 mg/ml) were dispersed in 5 mL of the chitosan solution with sonication, during which the chitosan was electrostatically attached to the MNP surfaces. Aggregation of MnFe_2_O_4_ nanoparticles was not observed during the reaction, indicating that DMSA-capped MnFe_2_O_4_ (DMSA-MnFe_2_O_4_) nanoparticles were stable under the experimental conditions. 2.0 mL of an aqueous EDC solution (varied from 0.3 to 1.2 wt % (0.006–0.024 g of EDC)) was added with sonication for 4 h and reacted for 24 h at room temperature to crosslink the chitosan and fix the polymer-coated MNPs. EDC reacts with DMSA carboxyl groups on the surface of MnFe_2_O_4_ nanoparticles to form an amine reactive O-acylisourea intermediate. This intermediate reacts with an amine group on chitosan, yielding chitosan-MnFe_2_O_4_ MNPs. Finally, this sample was washed with water followed by magnetic separation. Thus, the positively-charged chitosan backbone is positioned on the negatively-charged surface of MnFe_2_O_4_ MNPs. While an electrostatic interaction between the negatively-charged DMSA and positively charged chitosan can be responsible for some of the polymer binding, we used EDC to form a strong bond that would ensure that the chitosan remains attached to the MNP and can function as a barrier to control drug release.

### 2.4. Characterization of Nanoparticles

The crystal structure of the nanoparticles was determined using a X-ray diffractometer (Rigaku model D/Max-2BX X-ray diffractometer) with CuKα radiation. The morphology and crystal lattice of the samples were determined using a field emission scanning transmission electron microscope (FEI Technai F20 STEM, 200 keV). Magnetic characterization was performed using a vibrating sample magnetometer (Digital Measurement Systems model 1600 vibrating sample magnetometer). The thermal stability of the chitosan-coated MNPs was measured on a Thermal Gravimetric Analyzer (TA Instruments model 2920) with a heating rate of 8 °C /min under nitrogen flow. FTIR spectra were obtained on a spectrometer (BIO-RAD model FTS-40 FT-IR) and used to confirm the presence of characteristic bands related to the successful binding of chitosan to the MNPs via the EDC reaction. A dynamic light scattering instrument (DLS, Photocor Complex, College Park, MD, USA) equipped with a 633 nm He-Ne laser determined particle size distributions in aqueous solutions. Samples were dispersed in dust-free DI water after syringe filtration (Osmonics, Inc., 0.4 micron, Minnetonka, MN, USA). DLS experiments were used to determine particle size as a function of EDC concentration, and also to study the effect of pH on the swelling and collapse of the chitosan-coated MNPs. It should be noted that the plain DMSA-coated particles were not exposed to the pH buffers, as the particle dispersions are not stable in certain phosphate buffer solutions, particularly those that are slightly acidic; only chitosan-coated MNPs (which are stably dispersed in buffer solutions) were tested.

### 2.5. Heating Profiles of MNPs and Chitosan-Coated MNPs

The AC magnetic field-induced heat generation was measured for MnFe_2_O_4_ and chitosan-coated MNPs when exposed to various fields for 15 min using custom-designed magnetic- induction hyperthermia coils. The nanoparticles were dispersed in DI water at 0.349 wt %. The heating equipment is composed of a power supply (Nova Star 5kW RF Power Supply, Ameritherm, Inc, Scottsville, NY, USA), heating station and coils (Induction Atmospheres, Rochester, NY, USA), and chiller (Koolant Koolers Inc, Kalamazoo, MI, USA). The magnetic field was controlled by adjusting the voltage in the power supply. The temperature of MNP dispersions exposed to the AC magnetic field was measured using an IR camera (Thermacam^®^ SC2000, FLIR systems, Boston, MA, USA) to avoid magnetic interference with thermocouples. The IR camera was calibrated with an alcohol thermometer before every measurement. The temperatures were measured by focusing the camera on the top surface of each sample. The heating profiles were investigated at a frequency of 266 kHz with a 653 Oe magnetic field. A control experiment of applying the magnetic field to DI water in the same sample holder was conducted to confirm that there was no nonspecific heating occurring (such as heat conduction from the metal coils to the sample, which was prevented by placement of a polymer foam insulating material between the coils and sample holder). Heating studies were repeated at least three to five times to determine statistical reproducibility. The specific absorption rates for the nanoparticle solutions were calculated by [28]:

SAR = *CdT/dt* (5)

where *C* is heat capacity, m_s_ is the mass of the solution, m_p_ is the mass of MNPs, and *dT* is the differential temperature increase in time *dt*. The heat capacity was calculated from:

*C* = (*W_MnFe_2_O_4__C_MnFe_2_O_4__* + *W*_water_*C*_water_) / (*W_MnFe_2_O_4__* + *W*_water_) (6)

where *W_i_* is the mass of component i. The heat capacity of dry MnFe_2_O_4_ nanoparticles was measured using a Modulated Differential Scanning Calorimeter (DSC, Q200, TA Instruments, Newcastle, DE, USA) using a 1 °C/min ramp.

### 2.6. Drug-loading in chitosan-MnFe_2_O_4_ nanoparticles

The drug-loading capacities of chitosan-MnFe_2_O_4_ nanoparticles with different hydrodynamic diameters were determined using theophylline as a model drug. Theophylline was chosen as a model drug, since it is stable in water (water solubility 8.3 mg/g at 25 °C), and is easily detected by UV absorption at 275 nm. Theophylline was mixed into an aqueous chitosan solution prior to the linking to the surface of the MNPs. The drug loaded into the chitosan by simultaneous partitioning and crosslinking to sequester the drug near the nanoparticle surface. In this way, the theophylline (which is a small molecular weight drug that is stable under the reaction conditions) could be dispersed evenly throughout the chitosan layer. Excess drug was removed from the surface by washing three times followed by magnetic separation. The MNPs were dispersed in DI water to begin drug release studies. Theophylline-loaded chitosan-MnFe_2_O_4_ MNPs were synthesized using 0.3, 0.6, 0.9 and 1.2 wt % EDC. To separate the released theophylline from the chitosan-coated MNPs, 0.5 mL of the solution was filtered through a centrifugal 50,000 kDa ultrafilter (Millipore) at 3500 rpm for 15 min. The theophylline concentration in the filtrate was determined by UV absorbance at 275 nm (Shimadzu UV-2401 PC, MD, USA), and a calibration curve for a range of known concentrations, according to Beer’s law. Drug loading efficiency, DE, was calculated by mass balance:

DE = (Mo-M_1_)/Mo (7)

where Mo is the initial mass of drug added to the MNP solution and M_1_ is the unloaded amount remaining.

## 3. Results and Discussion

**Figure 2:**
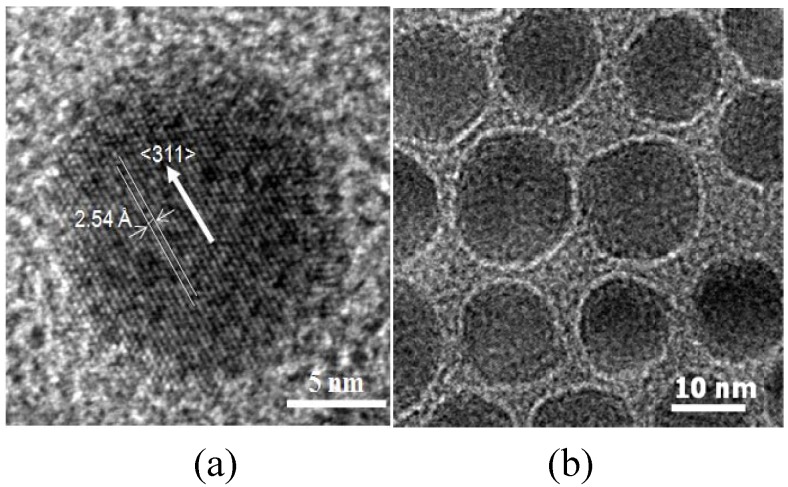
TEM images of the synthesized (a) MnFe_2_O_4_ MNPs in high-resolution and (b) chitosan-MnFe_2_O_4_ MNPs.

**Figure 3:**
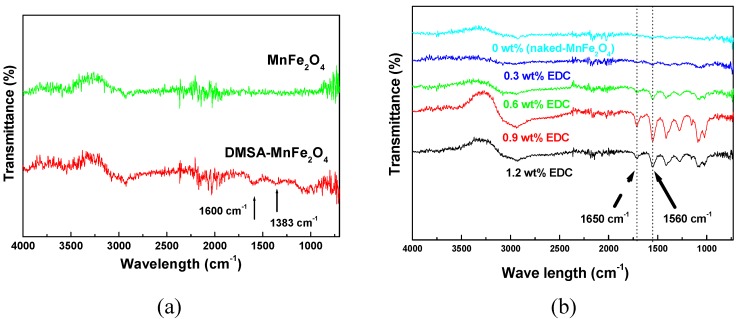
FTIR spectra of (a) DMSA-MnFe_2_O_4_ and (b) chitosan-MnFe_2_O_4_ MNPs.

MnFe_2_O_4_ MNPs were synthesized by seed-mediated growth. The MNPs had a spinel crystal structure as ascertained by XRD. The MNPs were nearly spherical with an average particle size of 12.1 nm (±0.4 nm) (Figure 2a). The HRTEM image (Figure 2a) of MnFe_2_O_4_ nanoparticles shows lattice fringes with the interfringe distance measured to be 2.54 Å, close to the lattice spacing of the <311> planes at 2.56 Å in the cubic spinel structure of MnFe_2_O_4_. The MNPs were superparamagnetic with a saturation magnetization, M_s_, of 44.1 emu/g. The chitosan coating on MNPs was characterized by TEM, FTIR, TGA and VSM. A layer of chitosan surrounding the core MnFe_2_O_4_ nanoparticles was observed in TEM images (Figure 2b), indicating, that while the acetic acid present during the reaction could have also been coupled to chitosan through EDC, the chitosan has been successfully attached to the surface of the MnFe_2_O_4_ NPs, forming a thin layer of approximately 1 nm. The chitosan-coated MNPs had characteristic FTIR peaks at 1650 and 1560 cm^-1^, corresponding to amide I and amide II bands, respectively (Figure 3) [29]. The height of these bands increased as the EDC used increased from 0.3 to 0.9 wt % but at 1.2 wt % EDC, these bands decreased to a level similar to 0.6 wt % EDC.

The DMSA-coated MNPs were stable in the pH range of 6-11. FTIR spectra confirm the bond of DMSA to MnFe_2_O_4_ MNPs, as bands at 1600 cm^-1^ and 1383 cm^-1^ were observed (Figure 3a), characteristic of the carboxylic acid-iron bond expected. DMSA is a dithiol (containing two sulfhydryl, or S-H, groups) and an analogue of dimercaprol. This is a water-soluble, non-toxic, metal chelator which has been administered orally as an antidote to heavy metal toxicity since the 1950s [30,31]. Therefore, MnFe_2_O_4_ MNPs dispersed in water using DMSA have great potential for use *in vivo*.

**Figure 4:**
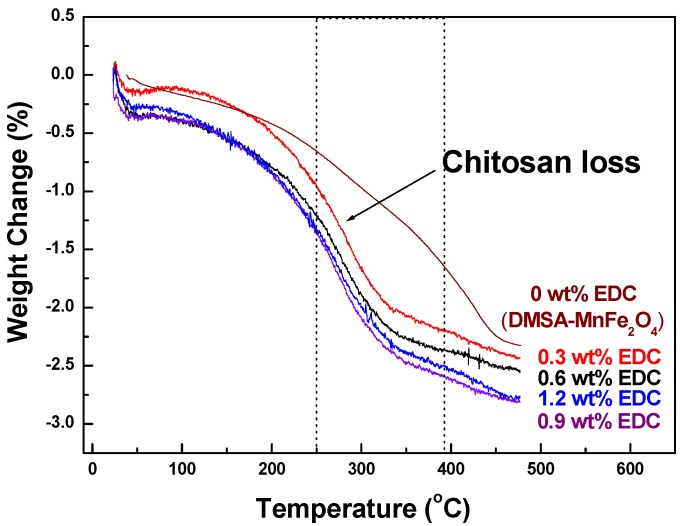
TGA curves of chitosan-MnFe_2_O_4_ MNPs synthesized using a range of EDC linker.

TGA thermograms measured the fraction of organic material (chitosan) on the MNPs. (Figure 4). For MNPs with no chitosan, the weight of samples gradually decreased between room temperature and 440 °C. However, the characteristic weight loss of chitosan-MnFe_2_O_4_ MNPs started at 220 °C and continued up to 340 °C, during which there was from 2.03% to 2.39% weight loss due to the degradation of chitosan. The highest mass loss due to chitosan was seen for coated MNPs made with 0.9 wt % EDC, confirming the FTIR data.

**Figure 5:**
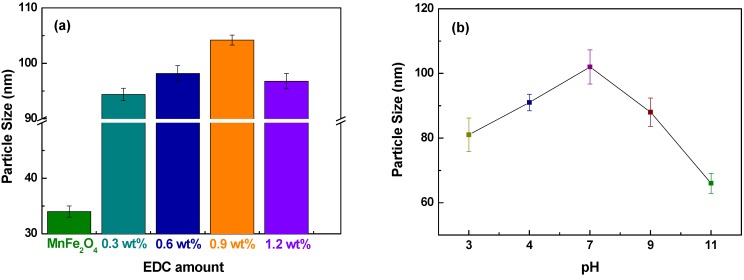
Average hydrodynamic size of chitosan-MnFe_2_O_4_ MNPs dependent on (a) the EDC concentration used during chitosan coating in DI water and (b) pH-dependent average hydrodynamic size of chitosan-MnFe_2_O_4_ MNPs at 0.9 wt % EDC. Error bars represent the standard deviation for three replicates. pH buffers (Metrepak pHydrion buffers, Brooklyn, NY) used include: pH 3 and 4: 0.05 M potassium biphthalate pH 7: 0.05 M phosphate; pH 9: 0.04 M sodium bicarbonate/sodium carbonate and pH 11: 0.03 M sodium bicarbonate/sodium carbonate.

The average sizes of chitosan-MnFe_2_O_4_ MNPs determined by DLS were much larger than observed using TEM, as also shown by Lopez-Cruz *et al.* [32]. The average size of chitosan-MnFe_2_O_4_ nanoparticles determined by TEM was approximately 18 nm but the diameter of chitosan-MnFe_2_O_4_ nanoparticles determined by DLS ranged from 94.4 to 104.2 nm, showing aggregation that is common in aqueous nanoparticle systems. In TEM, the chitosan-coated MNPs were spread out in a thin film and dried prior to measurement, while DLS measured particle size in solution. Thus, TEM was capable of distinguishing individually-coated MNPs, while DLS measured the aggregation of MNPs in solution. The hydrodynamic size of chitosan-MnFe_2_O_4_ was strongly dependent on the quantity of EDC used to attach chitosan (Figure 5a). The hydrodynamic size of the samples increased from 94.4 to 104.2 nm of diameter as the EDC crosslinker was increased from 0.3-0.9 wt %.

Because chitosan has multiple amino groups, it is sensitive to the pH of the solution. It can ionize in buffered solutions, and has been shown to attach to DMSA-MNPs through electrostatic interactions [33]. In our study, EDC was used as an additional linking agent to build a stronger bond between the MNPs and chitosan. In addition, sonication during the linking reaction broke the ionic interactions, to ensure that chitosan was linked to the MNPs through EDC. EDC reacts with a carboxyl to form an amine reactive O-acylisourea intermediate. If the intermediate does not encounter an amine, it will hydrolyze and regenerate the carboxyl group. Therefore, the amount of chitosan coating was limited by the amount of carboxyl groups on the DMSA-MnFe_2_O_4_ nanoparticles. Thus, 0.9 wt % EDC was optimal for maximizing chitosan attachment (Figure 5a). As has been shown by Lopez-Cruz *et al.* [32], the presence of chitosan bound to the MNP surface help to stabilize the dispersion when in the presence of buffer ions, particularly phosphates. Here, we investigated the hydrodynamic particle size as a function of pH, as a potential way to trigger drug release. The average hydrodynamic diameter of the chitosan-MNPs was largest at pH 7 (Figure 5b). A reduced electrostatic repulsion between the chitosan chains around the pK_a_ of 6.5 [34] allowed greater agglomerations at neutral pH values despite chitosan being more expanded on a single nanoparticle when in acidic or basic solutions, as expected from swelling experiments on chitosan hydrogels [35]. This pH-responsive behavior of chitosan makes it potentially useful in a pH-triggered drug delivery system.

**Figure 6:**
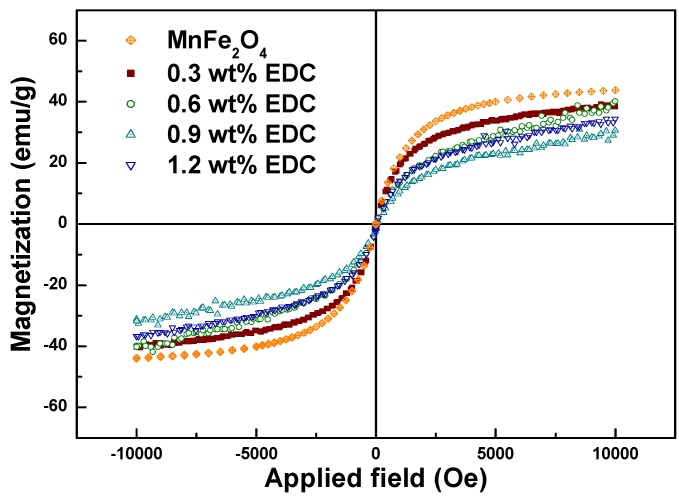
Magnetization curves for the DMSA-MnFe_2_O_4_ and chitosan-MnFe_2_O_4_ MNPs formed using different amounts of EDC.

Magnetization values of chitosan-MNPs decreased slightly as the amount of EDC was increased (Table 1). As shown in the FTIR, TGA and DLS analysis, the amount of EDC used to link chitosan to the MNPs controlled the coating thickness. Since the magnetization was normalized by the total sample weight, it decreased from 40.2 to 31.2 emu/g as the chitosan coating thickness increased (for EDC amounts ranging from 0.3 to 1.2 wt %) with the M_s_ of chitosan-MnFe_2_O_4_ nanoparticles synthesized using 0.9 wt % EDC 29.2% lower than that for uncoated MnFe_2_O_4_ MNPs (Figure 6). The decrease of total magnetic moment for the chitosan-coated MNPs is likely due to a non-collinear spin structure originating from the pinning of the spins by polymer coating at the MNP surface [36].

**Table 1:** Properties of the synthesized MnFe_2_O_4_ nanoparticles. Values for SAR are reported for a 7 mg/mL or 0.349 wt % aqueous nanoparticle solution. Reported errors represent the standard deviations for three replicates.

Sample	EDC Linker Concen- tration	TEM Particle Diameter (nm)	DLS Particle Diameter (nm)	Magnetization at 10 kOe (emu/g)	SAR (W/g) at 653 Oe, 266 kHz
DMSA- MnFe_2_O_4_	-	12.1 ± 0.4	34 ± 1.2	44.1	1.45
Chitosan- MnFe_2_O_4_	0.3 wt %	17.2 ± 1.3	94.4 ± 2.6	40.2	1.20
	0.6 wt %	17.7 ± 2.1	98.2 ± 3.2	39.8	1.17
	0.9 wt %	18.3 ± 1.6	104.2 ± 2.4	31.2	1.08
	1.2 wt %	17.1 ± 1.3	96.8 ± 2.2	36.7	1.15

**Figure 7:**
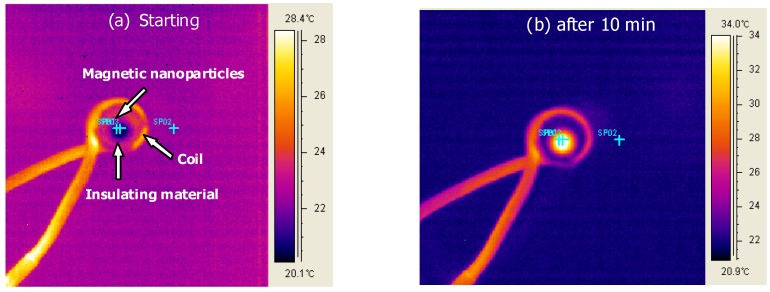
FLIR Thermacam^®^ images taken (a) at starting and (b) after 10 minutes for the heating of magnetic nanoparticles under a 653 Oe (266KHz) field.

The water-dispersed DMSA-MnFe_2_O_4_ nanoparticles, both with and without chitosan, were successfully heated using an AC magnetic field. In an earlier study [35], heating of CoFe_2_O_4_ MNPs was controlled by the amplitude and frequency of an AC magnetic field. Here, the effect of a polymer coating on the MNPs was investigated. Images collected from the FLIR Thermacam^®^ show the experimental set-up and were used to measure the magnetically-induced heat generation (Figure 7).

**Figure 8:**
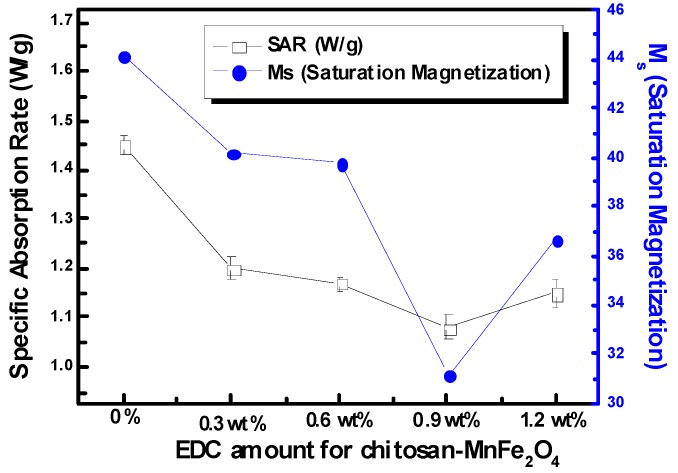
SARs and M_s_ (saturation magnetization) of the chitosan-MnFe_2_O_4_ MNP solutions formed using various EDC amount at the concentrations of 7 mg/ml of the MnFe_2_O_4_ nanoparticles under a 653 Oe (266 kHz) field. Error bars for SAR represent the standard deviation for three samples. No error is reported for M_s_.

The SAR of the nanoparticle solutions was used as the standard measure of heating efficacy. Using modulated DSC, the heat capacity of MnFe_2_O_4_ was determined to be 0.857 J/g °C. The heat generated in aqueous solutions of chitosan-MNPs was lower than that for DMSA-MNPs at a concentration of 7 mg/ml (Figure 8). The solution SARs continued to drop as more EDC was used to coat chitosan onto the MnFe_2_O_4_ MNPs, largely following the trends observed for saturation magnetization. Although SAR is a standard variable for reporting magnetically-induced heating, it varies with nanoparticle concentration, as well as the applied magnetic field and frequency, making it difficult to compare data from different researchers. To the best of our knowledge, this is the first report of SAR values for MnFe_2_O_4_ MNPs, as this is a newer material for use in hyperthermia applications. While these values for SAR are considerably lower than for bulk nanoparticles, it should be noted that these values are for the heating of the solution and are not normalized with respect to just the MNP mass. To improve SAR, the concentration of the MNPs can be increased, and other parameters (nanoparticle size, and magnetic field frequency and intensity) can be optimized. Baker *et al.* [28] reported that the SAR of a one wt % Fe_2_O_3_ (9 nm) nanoparticle concentration in an epoxy resin for localized hyperthermia was 1.25 W/g at 300 kHz and 150 Oe. Here, an aqueous solution of DMSA-MnFe_2_O_4_ at 0.349 wt % was found to have a SAR of 1.45 W/g at 266 kHz and 653 Oe, which leads to strong possibilities for its use in hyperthermia cancer treatment which can be combined with the usefulness of MnFe_2_O_4_ as a phase contrast agent for imaging.

**Figure 9:**
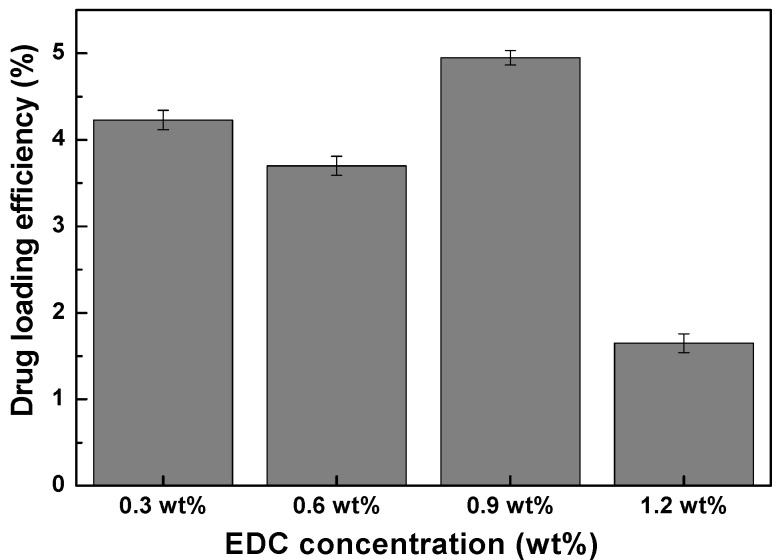
Drug-loading efficiency of the chitosan-MnFe_2_O_4_ MNP samples, as % of initial drug mixed with the MNPs that were effectively released. Error bars represent the standard deviation for three replicates.

The drug loading efficiency of chitosan-MnFe_2_O_4_ nanoparticles of different hydrodynamic sizes were measured using theophylline. Theophylline release was achieved and monitored by separating the supernatant theophylline (with molecular diameter 3.8 Å) released from the 18 nm chitosan-MnFe_2_O_4_ nanoparticles by centrifugal ultrafiltration at 3,500 rpm for 15 minutes. The drug-loading efficiency in chitosan-MnFe_2_O_4_ nanoparticles was calculated by comparing the amount of drug effectively released with the initial amount of theophylline used in the loading solution (Figure 9). The loading was shown to be most effective for the chitosan-MnFe_2_O_4_ nanoparticles synthesized using 0.9 wt % EDC. Thus, the chitosan-MnFe_2_O_4_ nanoparticles with the thickest chitosan layer were shown to absorb the greatest amount of theophylline. For the chitosan-MNPs linked using 1.2 wt % EDC, even though the chitosan layer was observed to be substantial (Figure 5), the additional crosslinking may have prevented effective uptake of the model drug theophylline.

## 4. Conclusions

Chitosan-coated MnFe_2_O_4_ nanoparticles were successfully synthesized as a multifunctional magnetic nanoparticle with the potential to combine localized heat generation for hyperthermia treatment with a carrier for drug delivery and a MRI contrast agent. The as-synthesized hydrophobic MnFe_2_O_4_ nanoparticles were changed to hydrophilic using DMSA ligand exchange to successfully disperse in water. Chitosan was coated on the surface of MnFe_2_O_4_ nanoparticles using EDC as a linking agent. The size of chitosan-MnFe_2_O_4_ nanoparticles was controlled by the concentration of EDC used. The AC field-induced heating of chitosan-MnFe_2_O_4_ nanoparticles was demonstrated using infrared imaging. Compared 1.45 W/g of uncoated MnFe_2_O_4_, the SARs of chitosan-MnFe_2_O_4_ nanoparticle solutions were somewhat reduced (1.08 to 1.2 W/g). In addition to this, our results show that a model drug can be incorporated in the chitosan layer for later release. The amount of drug that could be loaded into the chitosan-MnFe_2_O_4_ nanoparticles was controlled primarily by the quantity of EDC used for linking that led to different chitosan coating thicknesses. The most important findings in this work are that (1) manganese ferrite nanoparticles can be made and dispersed in aqueous solutions, (2) that these nanoparticles display significant heating for hyperthermia applications (which when adds to their already-reported use in MRI), (3) nanoshells of chitosan can be grafted to the surface of MnFe_2_O_4_ nanoparticles and used to sequester a model drug. Thus, we hope that this work will enable the further development of a multifunctional platform for imaging and dual-use therapy.
